# Evaluation of parallel endplate osteotomy for severe rigid spinal deformities: a retrospective analysis of 36 cases with a minimum 2-year follow-up

**DOI:** 10.1186/s12891-021-04877-3

**Published:** 2021-12-03

**Authors:** Hang Liao, Peng Xie, Guizhou Zheng, Houguang Miao, Ningdao Li, Xuedong Li, Shixin Du

**Affiliations:** Department of Orthopedics, The Third Affiliated Hospital (The Affiliated Luohu Hospital) of Shenzhen University, Shenzhen, 518000 PR China

**Keywords:** Severe rigid spinal deformities, Parallel endplate osteotomy, Clinical outcomes, Spinal cord safety, Complications

## Abstract

**Background:**

To report on the technique and results of parallel endplate osteotomy (PEO) for severe rigid spinal deformity.

**Methods:**

We retrospectively reviewed the clinical data of 36 patients with severe rigid spinal deformities who underwent PEO between July 2016 and December 2018 and who were followed up for at least 24 months.

**Results:**

Following PEO, the kyphosis and scoliosis correction rates reached 77.4 ± 14.0% and 72.2 ± 18.2%, respectively. The median intraoperative estimated blood loss was 1500 mL and the median operative time was 6.8 h. The SF-36 scores of physical function, role-physical, bodily pain, general health, vitality, social function, role-emotional and mental health changed from 62 ± 28, 51 ± 26, 49 ± 29, 35 ± 30, 53 ± 28, 45 ± 30, 32 ± 34 and 54 ± 18 at baseline to 81 ± 16, 66 ± 41, 72 ± 40, 64 ± 44, 75 ± 25, 71 ± 46, 66 ± 34 and 76 ± 28 at 12 months postoperatively, 82 ± 32, 67 ± 42, 81 ± 30, 71 ± 41, 80 ± 30, 74 ± 36, 68 ± 35 and 85 ± 33 at 18 months postoperatively, and 86 ± 21, 83 ± 33, 88 ± 26, 79 ± 39, 86 ± 36, 86 ± 48, 80 ± 47 and 91 ± 39 at 24 months postoperatively, respectively.

**Conclusions:**

PEO is an effective technique for successful correction of spinal deformities. At the two-year follow-up visit, all patients achieved better clinical results based on the SF-36 scores.

## Background

**Fig. 1:**
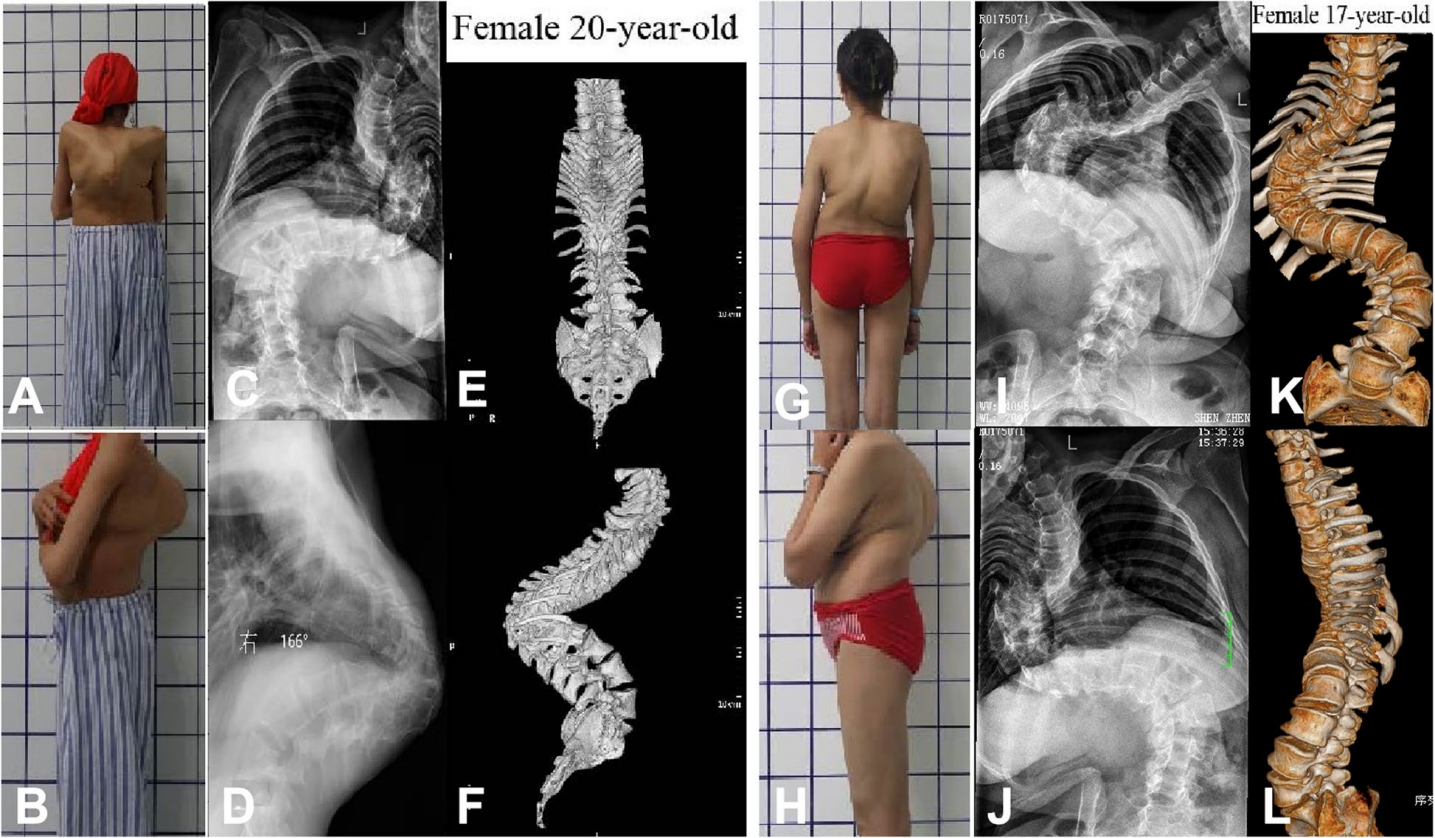
Female, 22 years old, severe angular kyphosis. **A**, **B**, **C**, **D**, **E** and **F** Preoperative diagnosis was kyphosis: Cobb angle 166° by profile, X-ray after bending, three-dimensional CT. Female, 17 years old, **G**, **H**, **I**, **J**, **K** and **L**) Preoperative diagnosis was kyphosis: Cobb 99° and scoliosis Cobb 97° by profile, X-ray after bending, three-dimensional CT

Severe, rigid angular kyphosis (Fig. 1A, B, C, D, E and F) and kyphoscoliosis (Fig. 1G, H, I, J, K and L) were considered insurmountable challenges for spinal correction surgery in the last century. Despite tremendous efforts by spine surgeons to find effective solutions, the corrective outcomes remain unsatisfactory, with a high incidence of neurological complications and massive bleeding, posing major threats to various spinal correction procedures and technologies.

In severe spinal deformities, the Cobb angle of the main curve is more than 80–100°; in rigid spinal deformities, the flexibility of the main curve is less than 10–30 % [1]. Treatment is often accompanied by difficulties and complications. Many studies have identified a relationship between anterior thoracotomy and a further decline in the pulmonary function of the patients; therefore, surgeons prefer a posterior approach alone [2–4].

Although different types of osteotomies have been described and widely used to address large, stiff scoliotic or kyphotic curves [5–11], it should be noted that abnormal pedicle development, including absence of pedicles, cause more difficulties in osteotomies. However, the Scoliosis Research Society Morbidity and Mortality Committee reported that the incidence of complications including neural and non-neural injury during Smith Peterson osteotomies (SPO), pedicular subtraction osteotomy (PSO) and vertebral column resection (VCR) was 28.1, 39.1 and 61.1%, respectively [12, 13]. To overcome these adversities, the present study was undertaken to evaluate and report on the technique and outcomes of parallel endplate osteotomy (PEO) for severe rigid spinal deformities at a single tertiary care institution.

## Methods

### Patients

This retrospective study enrolled patients with severe rigid spinal deformities who underwent PEO between July 2016 and December 2018. Spinal deformities were diagnosed by human grid analysis, roentgenography after bending or traction, three dimensional (3D) computed tomography (CT) and 3D printing models. The inclusion criteria were 1) a spinal scoliosis and kyphosis angle more than 80°; 2) flexibility less than 25%; 3) receipt of one-stage posterior-only PEO and correction surgery; 4) a minimum 2-year postoperative follow-up. We excluded patients with any neurological deficits including those with spinal cord or nerve root injury or other serious respiratory complications before surgery.

The study protocol was approved by the institutional review board of the authors’ affiliated institution. No patient consent was required because of the retrospective nature of the study. Patient data were anonymized in the paper.

### Assessment of deformity

Instrumentation levels were determined according to the Cobb angle and flexibility of the main curve. Severe rigid spinal deformities were defined as having curve angles more than 80°, with flexibility less than 25% by X-rays after bending or traction [14]. The site of osteotomy was usually chosen as the vertebra that contributed most to the deformity according to the apex of the deformity.

### Surgical technique

All surgeries were performed as a single-stage procedure by a single surgeon (the corresponding author, SXD) through a posterior incision. The surgical procedure has been described in our previous paper [15].

The patient was placed in the prone position on the operating table, on chest rolls. A single midline posterior longitudinal incision was made to expose the area at the previously determined levels. Paraspinal muscles and all soft tissues were stripped subperiosteally from the bone laterally to the tips of the transverse processes. Then, the pleural and paravertebral vessels were bluntly dissected. An intraoperative radiograph with guide pins was obtained for accurate localization of the deformity and determination of the level and area for osteotomy. Pedicle screws were inserted in the cephalic and the caudal aspects of the vertebrae identified for resection using a free-hand technique at all levels planned prior to surgery. It should be noted that abnormal pedicle development, including absence of pedicles, causes more difficulties in establishing the screw trajectory, and that screw insertion is time consuming [16]. Usually, the spine is stabilized with a short-bent rod in situ adjacent to the resected area to avoid coronal and sagittal plane translation during the reduction maneuver. Complete laminectomy and facetectomy were performed to expose the spinal cord. In the thoracic spine, the rib heads were removed to allow complete resection of the lateral wall of the vertebral body and to allow untethered motion of the vertebral column. The spinal cord is usually located in the concave curve side, but occasionally located in the convex curve side. In the latter case, we need to be more careful to avoid causing neurological complications due to high tension of the spinal cord. For example, some patients have no dural sac in the spinal cord, and in other cases the spinal cord is as tight as a cord with the diameter of only one-third of a normal spinal cord. Any slight maneuver would cause action potentials to decline sharply by over 50%, or even disappear. Timely identification and prompt intervention must be performed, including enlarging the resected area to reduce the tendency of the spinal cord abrupt turning.

**Fig. 2:**
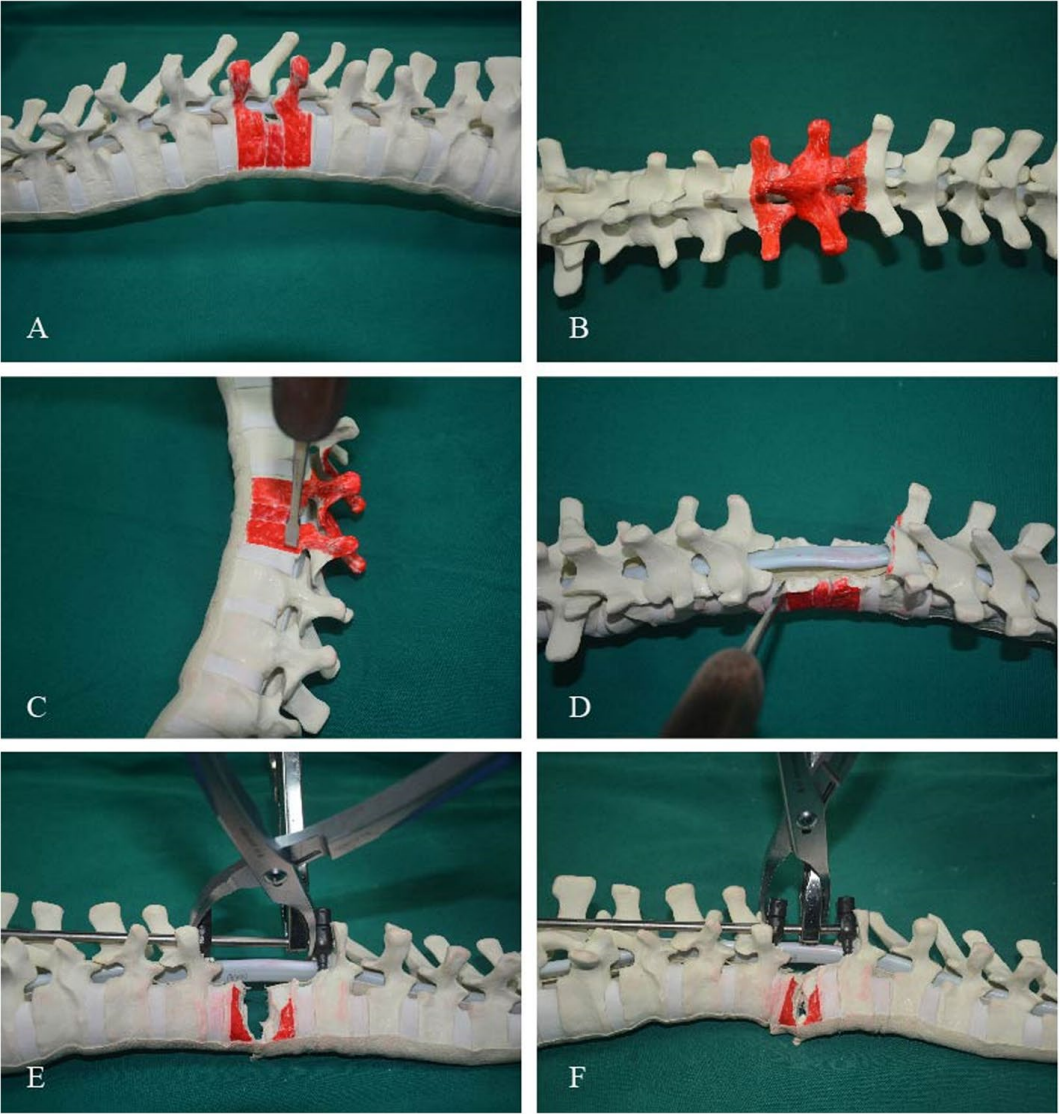
Simple operative schema of the thoracic parallel endplate osteotomy (PEO) procedure. **A**, **B** and **C** The resection region (the pedicle of the vertebral arch, 4/5 of the posterior vertebra, the bilateral walls of the vertebra and the posterior wall of the vertebra) is marked. **D** The temporary stabilizing rod is placed to the concave side to complete spinal canal decompression and PEO is performed. **E** and **F** Compression over the resected area and shortening of the spine are performed to reduce tension on the spinal cord. The surgeon then bends the rod in situ on the coronal plane or sagittal plane for correction and observes the migration of the dural sac; compression and bending of rods on both the convex and concave sides are undertaken to adjust tension of the spinal cord

**Fig. 3:**
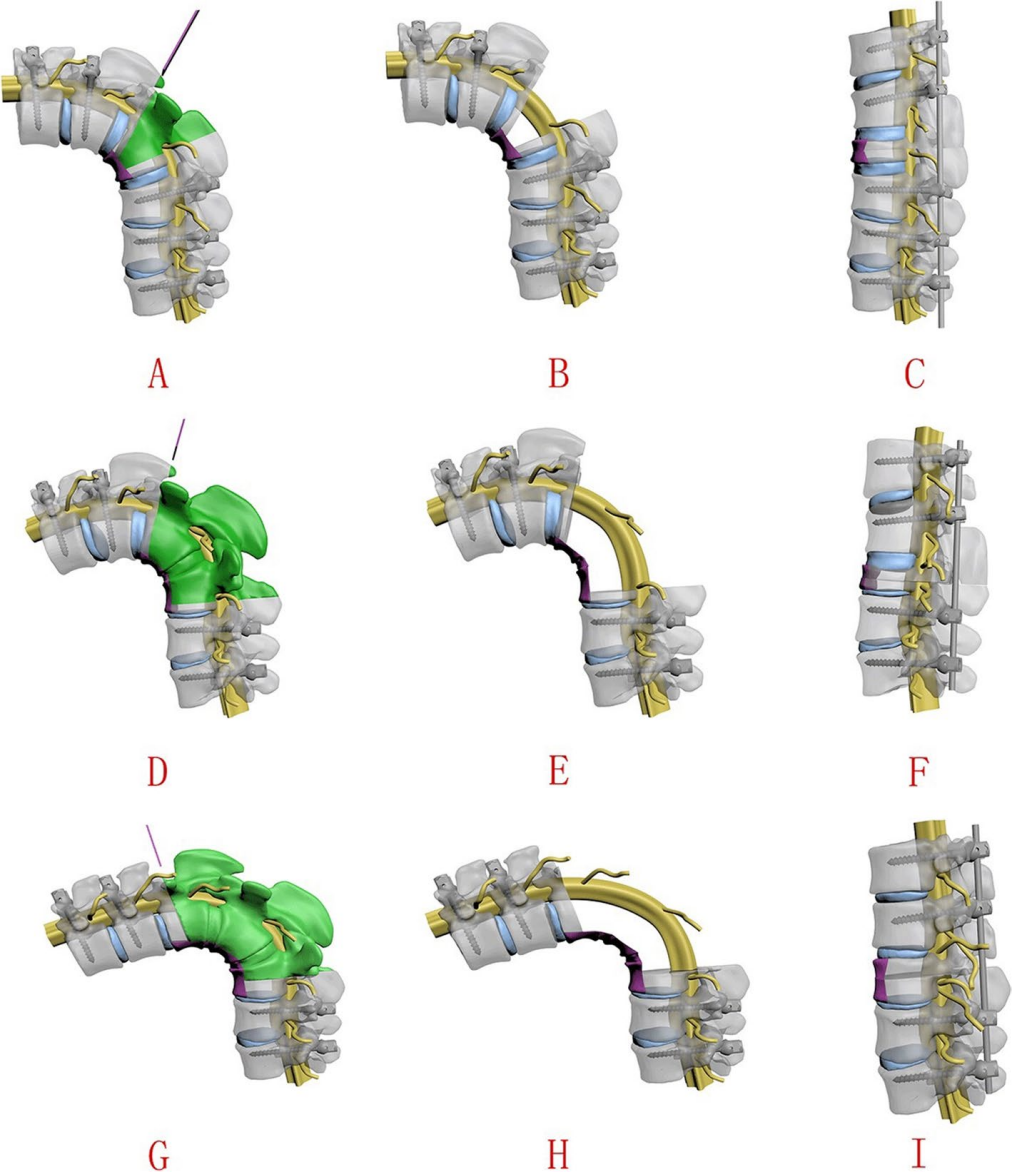
The 3-D digital demonstrations. **A**, **B** and **C** A single vertebral PEO. **D**, **E** and **F** The double-vertebra PEO. **G**, **H** and **I** The three-vertebra PEO

The spinal nerves were carefully dissected and preserved, but if they obstructed the osteotomy, one level of spinal nerve roots of the thorax on the convex curve side was usually resected. For PEO, the pedicle of the vertebral arch, 4/5 of the posterior vertebra, the bilateral walls of the vertebra and the posterior wall of the vertebra (5 mm to the endplate) (Fig. 2A-D) were carefully removed using an osteotome, curette, rongeur and ultrasonic osteotome. The apex area of PEO was planned in which the anterior 1/5 of the vertebral body was preserved during osteotomy. Compression over the resected area and shortening of the spine were performed to reduce tension on the spinal cord (Fig. 2E and F). The PEO area had two situations: 1) a single vertebral osteotomy, if the angle of the curve was less than 100° (Fig. 3A-C), and 2) a multiple vertebral osteotomy, if the angle of the curve greater than 100° (Fig. 3D-I).

The osteotomy was performed carefully to avoid over-penetration of the anterior vertebral body cortex or anterior intervertebral disc, for the purpose of providing a hinge point to avoid coronal and sagittal plane translation, and also to prevent injury to the major vessels in front of the vertebral body. Then, we inserted another pre-contoured correction rodon on the convex side to exchange the rods, with 30° per correction. It was important in this step to keep an adequate compression force on the concave rod while its adjunct screws on the cephalic side were slightly released until the concave rod and screws were tightened one by one. In situ rod bending on the concave side should never be performed because it is a very dangerous procedure to the naked spinal cord and applying too much torsion to the pedicle screws could easily cause screw loosening and rod bender to stick out and injure the spinal cord. Therefore, we did not use the bent bar in PEO. After repeated compression and shuttling the segmental transient rod, we finally placed the terminal fixation rods after the main correction was achieved. The temporary rods should be exchanged with new rods because their mechanical integrity may be impaired by bending. Then, segmental derotation, compression, and distraction on the secondary curves were performed to achieve final correction. During the entire correction procedure, the dural sac was closely observed to avoid migration in any direction, and tension of the spinal cord was assessed by observation and frequent palpation. Adequate and quick adjustments were needed to ensure that spinal cord tension does not exceed the initial state under distraction, and to prevent excessive kinking of the dural sac after spinal shortening. Kawahara et al. confirmed that the spine shortened within one-third of the height of the vertebrae did not lead to a functional change of the spinal cord [17], but we did not worry about the excessive ruga of the dural sac. Spinal stability is always carefully maintained by the pedicle screw-rod system to prevent sudden migration of the spinal cord due to unstable instrumentation. We placed the terminal fixation rods after the main correction was achieved. After completion of resection and deformity correction, we filled any residual gap with resected vertebral body bone morsels [18].

We monitored somatosensory-evoked potential (SEP) and motor-evoked potential (MEP) to effectively monitor the spinal cord and nerve roots under the supervision of an experienced neurophysiologic physician throughout the PEO procedure, and an additional wake-up test was performed after completion of the correction step at the end of the surgery assess to the neurological status.

#### PEO grade

**Fig. 4:**
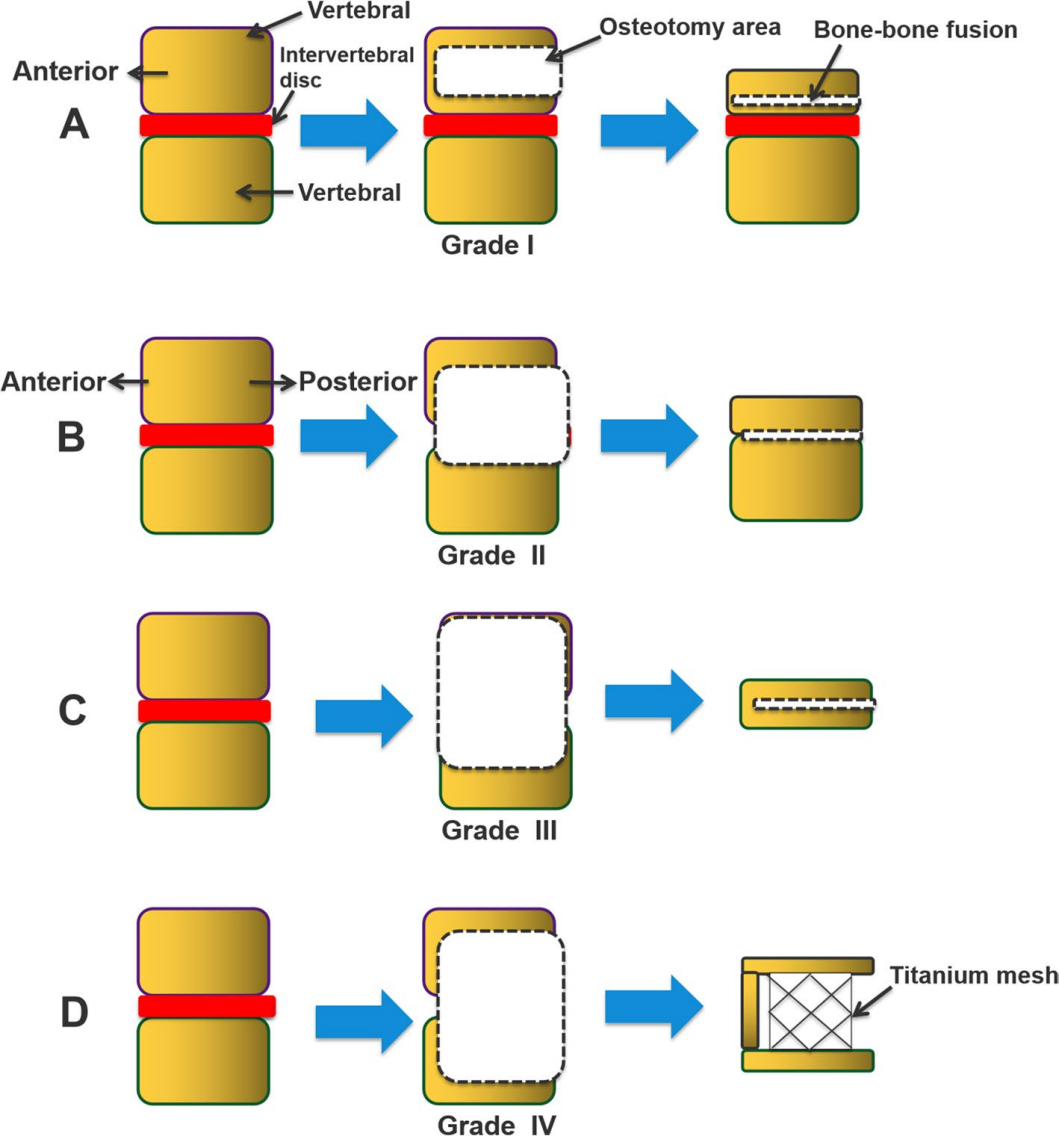
The PEO osteotomy grade classification. **A** Grade I: For scoliosis and kyphosis angle less than 80°, PEO with a single vertebra is recommended. **B** Grade II: For scoliosis and kyphosis (80°-100°), PEO with a single vertebra and the intervertebral disc is recommended. **C** Grade III: For scoliosis and kyphosis (101°-120°), PEO with two or more vertebrae and the intervertebral disc is recommended. **D** Grade IV: For scoliosis and kyphosis angle more than 120°, PEO with two or more vertebrae and the intervertebral disc is recommended. According to the severity of spinal cord folds, blood supply, tolerance of spinal cord twists, electrophysiological monitoring or wake-up experiments are used to determine whether to add titanium mesh implants for the front support of the vertebra

**Fig. 5:**
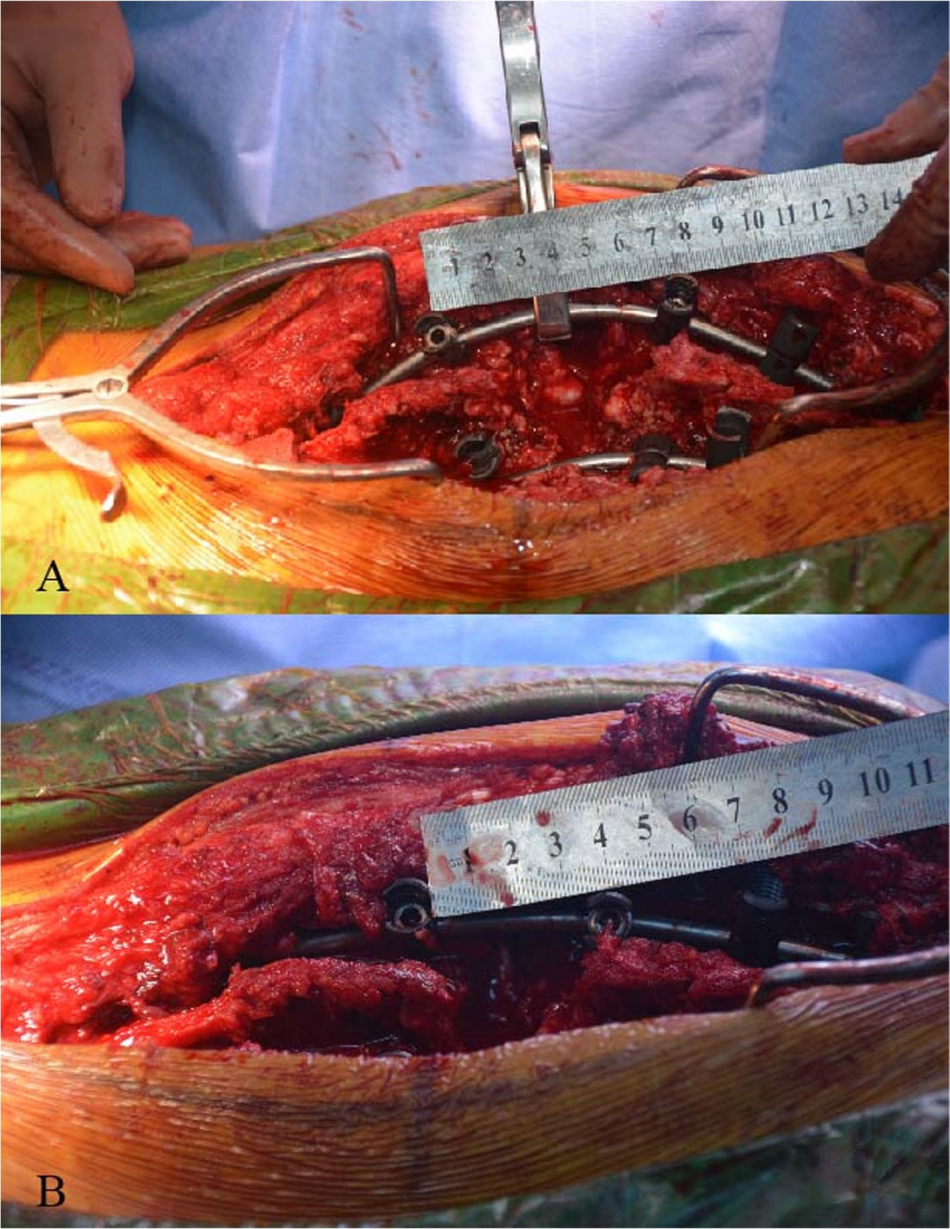
**A** and **B** Spinal shortening during PEO

Grade I: For patients with a scoliosis and kyphosis angle less than 80°, we recommended osteotomy that involved a single vertebra by PEO (Fig. 4A). The osteotomy angle can reach 50°-60°, and the correction rate can reach 80–85% by data from the previous cases. Grade II: For patients with a scoliosis and kyphosis angle between 80° and 100°, we recommended osteotomy that involved a single vertebra and the intervertebral disc by PEO (Fig. 4B). The osteotomy angle can reach 70°-85°, and the correction rate can reach 70–85%. Grade III: For patients with a scoliosis and kyphosis angle between 101° and 120°, we recommended osteotomy that involved two vertebrae and the intervertebral disc by PEO (Fig. 4C). The osteotomy angle can reach 80°-100°, and the correction rate can reach 70–85%. Grade IV: For patients with a scoliosis and kyphosis angle more than 120°, we recommended osteotomy that involved two or more vertebrae and the intervertebral disc by PEO (Fig. 4D). The osteotomy angle can reach 100°-120°, and the correction rate can reach 70–75%. According to the severity of spinal cord folds, blood supply, tolerance of spinal cord twists, electrophysiological monitoring or wake-up experiments to determine whether to add the titanium mesh implants for the front support of the vertebral, we had a maximum spinal shortening of 5 cm (Fig. 5A and B). For some patients with a smaller scoliosis and kyphosis angle but have multi-vertebral malformation (butterfly vertebra or other deformities) that need to be removed, a higher grade level of PEO osteotomy was adopted.

### Data analysis

Data were displayed as mean ± SD unless otherwise indicated. Student’s *t* test was used to evaluate the differences after surgery. Statistical significance was set at a value of *P* < 0.05.

## Results

In total, 36 severe rigid spinal deformities patients received a PEO. The 3D model provided accurate diagnostic and better surgical options. The kyphosis and scoliosis correction rates reached 77.4 ± 14% and 72.2 ± 18.2%, respectively. The median intraoperative estimated blood loss was 1500 mL and median the operative time was 6.8 h (Table 1). The median duration of follow up was 32 months (range 24 to 50 months). Table 2 shows SF-36 scores of the patients at baseline, and at 12 months, 18 months and 24 months postoperatively, respectively. The SF-36 scores of physical function, role-physical, bodily pain, general health, vitality, social function, role-emotional and mental health changed from 62 ± 28, 51 ± 26, 49 ± 29, 35 ± 30, 53 ± 28, 45 ± 30, 32 ± 34 and 54 ± 18 at baseline to 81 ± 16, 66 ± 41, 72 ± 40, 64 ± 44, 75 ± 25, 71 ± 46, 66 ± 34 and 76 ± 28 at 12 months postoperatively, 82 ± 32, 67 ± 42, 81 ± 30, 71 ± 41, 80 ± 30, 74 ± 36, 68 ± 35 and 85 ± 33 at 18 months postoperatively, and 86 ± 21, 83 ± 33, 88 ± 26, 79 ± 39, 86 ± 36, 86 ± 48, 80 ± 47 and 91 ± 39 at 24 months postoperatively, respectively (*P* < 0.05, Student’s t test), indicating that the quality of life of the patients improved significantly after PEO.

**Table 1:** Summarized data of patients

Patient No.	Age/ Sex	Etiology	Kyphosis Cobb (degrees) Pre-op Post-op	Scoliosis Cobb (degrees) Pre-op Post-op	Osteotomy segments	Osteotomy grade	Upper and lower end vertebra	Bleeding (mL)	Operative time (hours)
1	33/F	Idiopathic	85 12	67 15	T11/T12	III	T8-L4	1800	5.6
2	22/M	Idiopathic	90 25	130 40	T12/L1	IV	T5-L5	3000	11
3	25/F	Congenital	112 20	98 18	T12/L1	III	T8-L5	1500	5.8
4	8/M	Tuberculosis	98 16	–	T10/T11/T12	IV	T5-L4	1000	6
5	21/M	Idiopathic	102 32	118 35	L1/L2	III	T8-S1	3000	5.5
6	10/F	Congenital	90 22	78 16	T11/T12	III	T6-L3	1500	5
7	15/M	Idiopathic	108 24	131 35	T9/T11/T12	IV	T4-L5	2600	9
8	24/F	Congenital	100 15	85 14	T12/L1	III	T9-L5	1800	6.3
9	16/M	Congenital	148 44	–	T12/L1	IV	T5-L4	2000	10
10	20/F	Congenital	166 46	–	T10/T11/T12	IV	T5-L5	1700	7
11	38/F	Idiopathic	85 25	63 15	L1	II	T9-L4	2500	5.5
12	32/M	Congenital	100 20	54 17	T12	II	T8-L4	1000	7
13	31/M	Idiopathic	–	91 29	T8	II	T4-L4	800	10.2
14	19/M	Idiopathic	–	97 25	T10	II	T5-L3	3500	10.4
15	22/F	Idiopathic	–	105 28	L2	II	T12-L3	2000	13.4
16	14/M	Idiopathic	–	91 24	T9	II	T5-L1	1400	5.8
17	12/M	Idiopathic	–	90 25	T10/T11	III	T4-L4	800	7
18	19/M	Idiopathic	–	100 24	T7	II	T5-L5	3000	9.3
19	19/F	Idiopathic	–	107 27	T12/L1	III	T5-L5	1500	6.8
20	42/F	Ankylosing	90 19	–	L3	II	T11-L5	1300	5.9
21	25/M	Ankylosing	85 15	–	L2	II	T8-L5	1500	6.3
22	43/F	Ankylosing	85 10	–	L3	II	T9-L5	1800	6.3
23	55/F	Congenital	82 12	–	L3	II	T7-L5	1500	5.3
24	42/M	Ankylosing	85 18	–	L2	II	T9-L5	1500	7
25	17/F	Idiopathic	99 20	97 21	T7/L2	III	T3-L5	1800	10.4
26	25/M	Idiopathic	108 28	131 40	T7/T8	IV	T8-L5	2600	8.8
27	16/F	Idiopathic	–	105 37	T5/T6	III	T3-L5	2000	9.3
28	14/M	Congenital	103 23	–	T9/T10	III	T5-L1	2100	6.8
29	38/F	Ankylosing	90 18	–	L2	II	T9-L4	1500	6
30	45/M	Ankylosing	85 15	–	L2	II	T10-L5	1000	6.8
31	8/M	Congenita	46 15	103 35	T9	II	T7-L2	600	4.8
32	11/F	Congenita	–	80 16	T4/T5	III	C5-T9	1000	6.5
33	14/M	Idiopathic	–	102 47	T8/T9	III	T4-L2	1300	8.8
34	13/F	Congenita	92 25	107 35	T9/T10	III	T5-L5	1500	8.8
35	38/F	Idiopathic	82 30	63 17	T10	II	T9-L4	2500	5.5
36	15/M	Idiopathic	–	147 60	T7/T8	IV	T4-L3	3500	9

**Table 2:** SF-36 scores before surgery and at 12, 18 and 24 months postoperatively. (*n* = 36)

Time points	Physical Function	Role-Physical	Bodily Pain	General Health	Vitality	Social Function	Role-Emotional	Mental Health
Before surgery	62 ± 28	51 ± 26	49 ± 29	35 ± 30	53 ± 28	45 ± 30	32 ± 34	54 ± 18
After surgery								
12 months	81 ± 16	66 ± 41	72 ± 40	64 ± 44	75 ± 25	71 ± 46	66 ± 34	76 ± 28
18 months	82 ± 32	67 ± 42	81 ± 30	71 ± 41	80 ± 30	74 ± 36	68 ± 35	85 ± 33
24 months	86 ± 21	83 ± 33	88 ± 26	79 ± 39	86 ± 36	86 ± 48	80 ± 47	91 ± 39

Although the clinical effect of the PEO technique was apparent, complications were unavoidable. L1 nerve root injury occurred in three (8.3%) patients, with intraoperative abnormal SEP and MEP wave forms. To further prove that the L1 nerve root was damaged intraoperatively, we performed a lower limb EMG postoperatively. The symptoms of L1 nerve root injury were significantly improved by pharmacotherapy with mannitol and methylprednisolone and nutritional neurotherapy support with monosialotetrahexosylganglioside. Meanwhile, two (5.6%) cases developed hemothorax, which was effectively repaired without any leakage, and a closed thoracic drainage tube was placed postoperatively. One (2.8%) patient experienced paralytic ileus which improved after gastric decompression, promoting intestinal motility and symptomatic medical treatment. One (2.8%) case developed superficial infection, which healed after thorough debridement. Two (5.6%) patients experienced distal screw loosening which improved after revision surgery. At the two-year follow up, we did not observe any other complications, such as dura laceration, nonunion/rod breakage and adjacent segment kyphosis (Table 3). At the last follow-up, firm bony fusion was observed in all patients.

**Table 3:** Complications in the study patients

Complication	Patients (*n* = 36)
Dura laceration	0
L1 nerve root injury	3 (8.3%)
Paralytic ileus	1 (2.8%)
Hemothorax	2 (5.6%)
Superficial infection	1 (2.8%)
Nonunion/rod broken	0
Distal screw loosening	2 (5.6%)
Adjacent segment kyphosis	0

### Typical case

#### PEO grade II

**Fig. 6:**
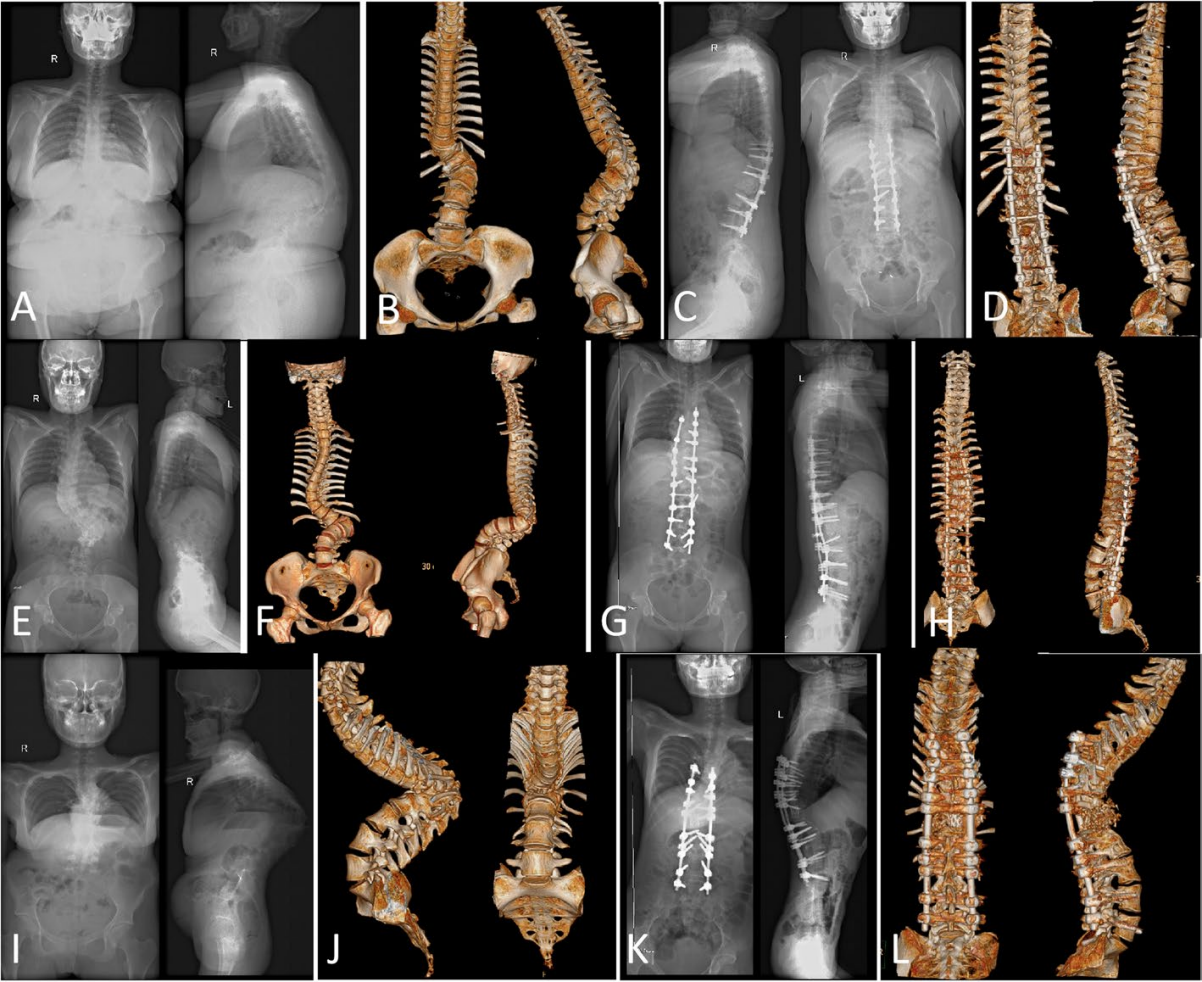
A 38-year-old woman with idiopathic kyphoscoliosis who received grade II PEO. **A** and **B** Preoperative reconstructed CT and radiographs showing scoliosis, Cobb (63°) and kyphosis, Cobb (85°). **C** and **D** Radiographs and reconstructed CT demonstrating the results of PEO with posterior spinal fusion; the scoliotic curve was corrected to 15° and the kyphotic curve to 25°. A 25-year-old woman with congenital kyphoscoliosis who underwent grade III PEO. **E** and **F** Preoperative reconstructed CT and radiographs showing scoliosis, Cobb (98°) and kyphosis, Cobb (112°). **G** and **H** Radiographs and reconstructed CT demonstrating the results of PEO with posterior spinal fusion: the scoliotic curve was corrected to 18° and the kyphotic curve to 20°. A 20-year-old woman with congenital kyphosis who received grade IV PEO. **I** and **J** Preoperative reconstructed CT and radiographs showing kyphosis Cobb, (166°). **K** and **L** Radiographs and reconstructed CT demonstrating the results of PEO with posterior spinal fusion and the titanium mesh implants for the front support of the vertebra; the kyphotic curve was corrected to 46°

A 38-year-old woman was diagnosed with idiopathic kyphoscoliosis (Patient No.11). Preoperative reconstructed CT scan and radiographs showed scoliosis (Cobb angle 63°) and kyphosis (Cobb angle 85°) (Fig. 6A and B). Postoperative radiographs and reconstructed CT scan demonstrated the results of PEO with posterior spinal fusion. The scoliotic curve was corrected to 15° and the kyphotic curve to 25° (Fig. 6C and Fig. 6D).

#### PEO grade III

A 25-year-old woman was diagnosed with congenital kyphoscoliosis (Patient No.3). Preoperative reconstructed CT and radiographs showed scoliosis (Cobb angle 98°) and kyphosis (Cobb angle 112°) (Fig. 6E and F). Postoperative radiographs and reconstructed CT revealed the results of PEO with posterior spinal fusion: the scoliotic curve was corrected to 18° and the kyphotic curve to 20° (Fig. 6G and Fig. 6H).

#### PEO grade IV

A 20-year-old woman with congenital kyphosis (Patient No.10). Preoperative photograph demonstrating reconstructed CT and radiographs showing Kyphosis Cobb (166°) planes (Fig. 6I and J). Radiographs and reconstructed CT demonstrating the results of PEO with posterior spinal fusion and the titanium mesh implants for the front support of the vertebral, the kyphotic curve was corrected to 46° (Fig. 6K and Fig. 6L).

## Discussion

Spinal deformity is a 3D deformity. Decompensation in the coronal and sagittal planes leads to specific complaints including pain, progression of deformity, deranged trunk balance, cardiopulmonary compromise, interference with daily living activities, and, in some cases, neurologic deficits [19]. The surgical objective for severe (Cobb angle > 80°) kyphosis and rigid spinal kyphoscoliosis deformities is decompression of neurological elements and correction of the deformities, which have always been a great challenge for spine specialists [20]. The surgical treatment is highly demanding and risky for both the surgeon and the patient [21]. In severe rigid spinal deformities, conventional correction methods, including posterior instrumentation and fusion or combinations of anterior release and posterior instrumentation and fusion, are usually unsatisfactory [22, 23]. Therefore, a more aggressive approach is necessary.

Posterior osteotomies allow correction through a hinge action. For severe rigid spinal deformities, the resection of apical region of the deformity is often performed by the PVCR procedure [24]. However, the hinge of the PVCR is the spinal cord, with the potential for various spinal cord-related neurological complications. PEO also differs from conventional vertebral column resection and PVCR in the usage of the spinal hinge, intraoperative deformity correction, and spinal reconstruction. However, the pedicle of the vertebral arch is taken as the main anatomic landmark of most surgical osteotomies. As for severe rigid spinal deformities, osteotomy is just inside the pedicle, which limits the range of osteotomy and leaves the orthopaedic surgeon unsatisfied. Those osteotomies are mostly PSO, VCD, eggshell and so on. PEO, which has been described in our previously published paper [15], includes the pedicle of the vertebral arch, 4/5 of the posterior vertebra, the bilateral walls of the vertebra and the posterior wall of the vertebra (5 mm to the endplate), which does not require thorough pedicular osteotomy like PSO and resection of the intervertebral disc above and below the osteotomy site like VCR. Moreover, the endplate as a mark in PEO is easy to identify which has a large operating space, and is especially suitable for pedicle deformity or agenesis which is unrecognizable, which is easier to operate for orthopedic surgeons. With bone-bone fusion, we can achieve a higher spinal fusion rate and better spine stability in order to reduce the risk of rod breakage. For spine deformity, PSO and VCR are classic techniques and can achieve satisfactory results. Meanwhile, the PEO technique provides a new alternative treatment for spinal deformities, with a median intraoperative estimated blood loss of 1500 mL and a median operative time of 6.8 h. The osteotomy angle can reach 110°-140°, basically satisfying any angle requirements for correction of spinal deformity. The kyphosis and scoliosis average correction rates reached 77.4 and 72.2%, respectively, which are better than the traditional correction rate of 55–60 % [25].

There is no permanent spinal cord injury in our series. The risk of spinal cord–related neurological complications is always emphasized in the literature concerning deformity correction. Potential neurological abnormalities should be noticed before corrective surgery. In our series of patients, 3/36 cases with very severe kyphoscoliosis had intraoperative neurological abnormalities. It was likely that the correction procedure caused spinal cord breakdown, thus increasing the tension on the spinal cord and reducing blood supply, accompanied by pathological changes. The patient presented with neurological symptoms intraoperatively, but operative exploration excluded mechanical compression, so the patient was treated with methylprednisolone and recovered completely within 2 weeks. Despite a different kind of anomaly, the corrective surgeries in these 3 patients achieved good results. We believe that a careful manipulation and continuous monitoring of spinal cord function are crucial for maintaining the integrity of neurological functions. In addition, severe deformity corrections that lead to spinal cord shortening can effectively relieve the over-tension of a tethered cord. In some cases of spinal deformity with multi-level vertebral loss, the spinal cord is in a state of high tension and has poor blood supply, minor traction during surgery can cause acute and severe spinal cord injury, leading to complete or incomplete paraplegia, these cases are considered to be high risk spinal cord cases. Though there is no definitive evidence as to what caused the neurologic compromise, we believe that it might be related to the blood supply of the thoracic cord and preoperative functional status of the spinal cord, which tolerates little additional compromise. At the two year follow-up after surgery, it is obvious from the SF-36 scores that all patients achieved better clinical results and with no other complications.

## Conclusions

We demonstrate that PEO is effective and relatively safe in correcting severe rigid spinal deformity. The PEO technique creates a hinge for spinal correction and spinal cord tension adjustment, and the correction can be performed under direct inspection and by observation and palpation of the tension in the spinal cord through the hinge. We propose for the first time the grade classification of PEO osteotomy. At the two-year follow-up after surgery, it is obvious from the SF-36 scores that all patients achieved better clinical results.

## Data Availability

All data generated or analysed during this study are included in this published article and its supplementary information files.

## Abbreviations

PEO: Parallel endplate osteotomy
SEP: Somatosensory-evoked potential
MEP: Motor-evoked potential
EMG: Electromyography
SPO: Smith Peterson osteotomies
PVCR: Posterior vertebral column resection
VCD: Vibrational circular dichroism
PSO: Pedicular subtraction osteotomy
VCR: Vertebral column resection
